# Development and validation of a novel and graded colour saturation threshold test, the LSS-8

**DOI:** 10.1186/s12886-026-04907-5

**Published:** 2026-05-30

**Authors:** Kristyna Stepnicka, Alexander Sarossy, Hannah McGrath, Marc Sarossy

**Affiliations:** 1 The University of Melbourne, Parkville, VIC, Australia; 2 Centre for Eye Research Australia, East Melbourne, VIC, Australia; 3 Barwon Health, Geelong, VIC, Australia; 4 Monash University, Clayton, VIC, Australia

**Keywords:** Color vision defects, Color vision, Color perception, Vision tests, Optic neuritis

## Abstract

Colour vision changes are a key clinical feature of many acquired retinal and optic nerve disorders. Current clinical tests are often labour-intensive or designed for congenital defects. We introduce the novel LSS-8 test, which measures colour saturation thresholds across eight hues. Three cohorts with mixed ocular health underwent testing in a diagnostic crossover study. The reliability cohort was tested with LSS-4 on different days (*n* = 30 eyes). The screen independence cohort used LSS-8 with 11-inch versus 13-inch screens in random order (*n* = 27 eyes). Brightness independence testing used LSS-8 at 115 cd/m^2^ versus 230 cd/m^2^ (*n* = 19 eyes). Scores for each plate set and total scores were recorded. Intraclass correlation coefficients (ICC) and difference frequency distributions were calculated. Data from 74 eyes (74 subjects; mean age 62.4 ± 19.1 years; range 20–85; 39% female) were analysed, with two participants contributing to two study phases. LSS-4 test-retest reliability with ICC(2,1) was good to excellent (mauve 0.93, red 0.95, green 0.87, blue 0.91, total score 0.96). LSS-8 screen independence testing showed excellent reliability for mauve, green, pink and total scores, and moderate to good reliability for other hues (ICC(2,k) 0.74–0.98). Varying screen brightness did not produce significant score differences in most participants. The LSS is a simple, reliable test with potential for rapid quantification of acquired colour vision defects.

## Introduction

Colour vision testing forms an important part of the diagnosis and monitoring of retinal and optic nerve diseases [1–4]. Colour vision defects may be congenital or acquired. Congenital dyschromatopsia typically results from inherited mutations affecting retinal cone photoreceptors and is generally bilateral and stable over time. In contrast, acquired dyschromatopsia may arise from a range of conditions including autoimmune disease, ageing, chemical toxicity, trauma and stroke, and is more variable in presentation [5, 6]. It may be asymmetric, progressive but potentially reversible, and may affect only one eye or a portion of the visual field. On conventional colour vision testing, congenital defects are usually present along the protan or deutan axes, whereas acquired defects are more commonly associated with tritan loss, although they do not always correspond neatly to a specific spectral region of colour discrimination loss [1, 6].

Acquired colour vision defects are associated with a wide range of conditions [1, 7–14]. For example, in retinitis pigmentosa, the deterioration of photoreceptors and the retinal pigment epithelium leads to colour deficiencies [8, 15]. In age-related macular degeneration (AMD) and diabetic retinopathy, choroidal hypoxia impairs both photoreceptors and the inner retina, disrupting colour perception [10, 16, 17]. For both optic neuropathy and glaucoma, colour vision deficits are related to retinal nerve fibre layer thinning and retinal ganglion cell death [7, 18]. Similarly, papilloedema in idiopathic intracranial hypertension can lead to optic nerve atrophy [19]. At the cortical level, cerebral achromatopsia, resulting from brain injury or posterior strokes, can impact colour perception more commonly than previously understood by clinicians [13]. Colour deficits have also been observed in patients treated with certain medications, for example hydroxychloroquine, clofazimine and ethambutol which cause retinopathy, maculopathy and optic neuropathy, respectively [6, 20–24]. Dyschromatopsia has also been reported in Parkinson’s disease and may precede the onset of motor symptoms [25].

Notably, for optic neuritis, retinitis pigmentosa, AMD, diabetic retinopathy and ethambutol toxicity, dyschromatopsia often precedes more severe symptoms. Acute optic neuritis is the most common optic neuropathy in young adults, with the incidence of unilateral optic neuritis ranging from 0.94 to 2.18 per 100,000 cases per year, and it is strongly associated with multiple sclerosis [7, 26]. Certain aggressive forms of optic neuritis, particularly those related to neuromyelitis optica (NMO) or myelin oligodendrocyte glycoprotein (MOG) antibodies are challenging to diagnose, lead to relapses and can result in permanent binocular vision loss [27, 28]. In these cases, early detection is critical for the administration of sight-saving treatments. Colour testing for sudden visual changes is therefore valuable in distinguishing optic neuritis from other visual pathologies where colour vision remains intact. It can also help track the progression or reversibility of the defect [27, 28]. In terms of medications, ethambutol and linezolid toxicity can lead to optic neuropathy [29, 30]. Clinical signs often present months after damage, however colour vision abnormalities are one of the first signs, supporting the role of colour vision testing as a screening tool for at-risk patients receiving these medications [22, 31].

In clinical practice, the Ishihara pseudoisochromatic plates are the most commonly used test for colour vision, designed to assess red-green congenital defects [6, 32]. One of the most comprehensive tests of colour vision is the Farnsworth-Munsell 100 hue (FM 100 hue). This test actually comprises 85 hues and is a labour-intensive arrangement test that assesses hue discrimination, specifically designed for detecting acquired colour vision losses [1, 33]. Error scores for each cap are calculated and plotted on a circular polar plot or the Munsell colour circle, highlighting areas of deficiency [34]. There are also computerised hue tests, for example the Seohan computerised hue test [35].

Other tests commonly used in clinical practice include the Farnsworth D15 test and Lanthony D15 test, both of which are rapid arrangement tests detecting severe abnormality [1]. The Farnsworth D15 is a shortened version of the FM 100 hue and was created to categorise individuals as having either deutan, protan or tritan defects [1]. The Lanthony D15 is a desaturated version of this and may also detect anomalous trichromacy [1]. The anomaloscope is considered the clinical standard for congenital colour blindness and is useful for the analysis of acquired colour vision defects yet it is still recommended to be combined with the FM 100 hue [1].

A well-known symptom in optic neuritis is subjective red desaturation, where patients report that red appears more “washed out” [36]. Efforts have been made to quantify this symptom [37–39]. Established colour vision tests that assess chromatic discrimination include the Hardy-Rand-Rittler (HRR) plates, which are primarily used for screening, the Mollon-Reffin Minimalist test, which uses coloured chips and requires examiner administration, and the Colour Assessment and Diagnosis (CAD) test which requires specialised equipment [40–42]. Littlewood described a test based on the Landolt C and the Ishihara plates that tested saturation threshold for a mauve hue and Bruegger and colleagues recently described a quantitative test of saturation threshold for a red hue [37, 38]. In this study, we extend the work of Littlewood to describe and validate a new test of colour vision. This new test, the Littlewood-Stepnicka-Sarossy test (LSS-8), determines the saturation threshold of colour perception for eight hues spaced around the Munsell colour circle and represents the results in a manner analogous to the FM 100 hue.

## Methods

### Participants

Three cohorts of individuals (74 subjects, total 74 eyes) with a mix of normal and abnormal ocular health were recruited at the Essendon Eye Clinic in Victoria from August 2023 to September 2024 inclusive. Each cohort was involved in a different phase of our study which included, in order: reliability (30 subjects, 30 eyes), screen independence (27 subjects, 27 eyes) and brightness independence testing (19 subjects, 19 eyes). Two participants each contributed the same eye to two study phases. We aimed to capture a general clinic population and therefore recruited agreeable individuals, without a strict inclusion criterion. This recruitment approach was deemed appropriate, given our aim for broad clinical applicability of the test. We excluded eyes which recently underwent surgery (≤1 month).

### Development

**Fig. 1:**
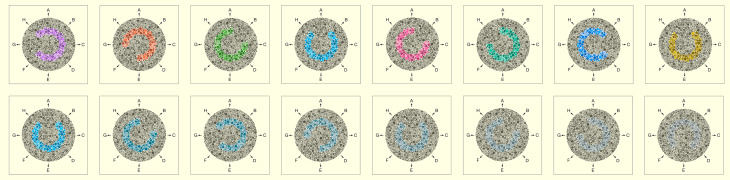
The LSS-4 and LSS-8 plate sets. In the upper row the first plate from each set of the LSS-8 is displayed, with the initial four plates of the top row representing the LSS-4 (mauve, red, green, and blue) and the remaining additional plates of the extended LSS-8 set shown in the next four plates (pink, teal, cobalt, and gold). The lower row illustrates the desaturation effect, featuring the first eight plates of the blue plate set

The LSS-8 is based on the C Test by Littlewood [37], which includes ten progressively more desaturated pseudoisochromatic plates in a single hue of mauve. Using Inkscape, C test colours were sampled to determine HEX codes. These codes were converted mathematically to the CIE LCH colour space where lightness (scored from 0 to 100), chroma and hue (0 to 360) could be manipulated [43]. In the LCH colour space, hues are represented by a colour wheel. When creating the LSS-4, hues at 90°, 180° and 270° from the original C-test hue were selected to create an additional three plate sets. An additional four plate sets were then developed to produce the complete LSS-8, with hues at 45°, 135°, 225° and 315° from the original C test. The LSS-8 therefore includes hues of the original mauve as well as red, green, blue (LSS-4) and pink, teal, cobalt, and gold. These hues were selected in the LCH colour space and showed approximate correspondence with positions on the FM 100 hue circle. Throughout this process, lightness and saturation were left unchanged from the original C test, though a novel random rotation of the C-shape occurred. For each hue, an additional 11th plate was created. Hue and lightness remained unchanged and chroma was reduced by 50%. Normalisation across the hues was performed as described below. During this development, for the gold set, chroma was increased by 25% for the first three plates. For the final two gold plates, the C-shape chroma corresponded to the 7th and 8th plates of the other sets although the background chroma paralleled the 10th and 11th plates. Notably, chroma, which is equivalent to saturation, is progressively reduced during testing using a descending method of limits until the detection threshold is reached. The plates underwent normalisation where 15 controls completed the test, scored similarly between hues, and reported comparable difficulty. The plate sets are shown in Fig. 1.

### Testing

Testing was performed monocularly. Subjects sat approximately 45 cm from the screen and were instructed to identify the gap in the C, which corresponded to eight positions, labelled A to H. Two attempts were permitted per plate and the score for a plate set was represented by the last plate number correctly identified. Each plate set was therefore scored out of 11 and the total score for the LSS-8 was out of 88.

During the test-retest reliability component, subjects were tested using the LSS-4 on separate occasions one week apart. For screen independence, subjects completed the test using an 11-inch iPad and 13-inch MacBook on the same day, in a random order. Similarly, for brightness independence, subjects completed the test on a screen with 115 cd/m^2^ and 230 cd/m^2^ on the same day in a random order. The room luminance was kept consistent between testing days. Participants were allowed to wear their glasses.

### Statistical analysis

The outcome measure was a participant’s score for each hue set and their total score. Statistical analyses were conducted using R. To calculate test-retest reliability, the intraclass correlation, ICC(2,1), was calculated using the psych package in R. ICC(2,k) was deemed more appropriate for calculating screen independence. For brightness independence, frequency distribution of differences was analysed.

## Results

### Demographic and clinical characteristics of participants

A total of 74 eyes from 74 participants were analysed in this study, consisting of 29 females and 45 males. The participants’ ages ranged from 20 to 85, with a mean age of 62.4 ± 19.1 years. The study included individuals with a variety of ocular health conditions, both normal and abnormal, such as optic neuropathy, glaucoma, cataracts, pseudophakic and phakic eyes, deuteranomaly, macular oedema and low vision.

### Flow of participants

**Fig. 2:**
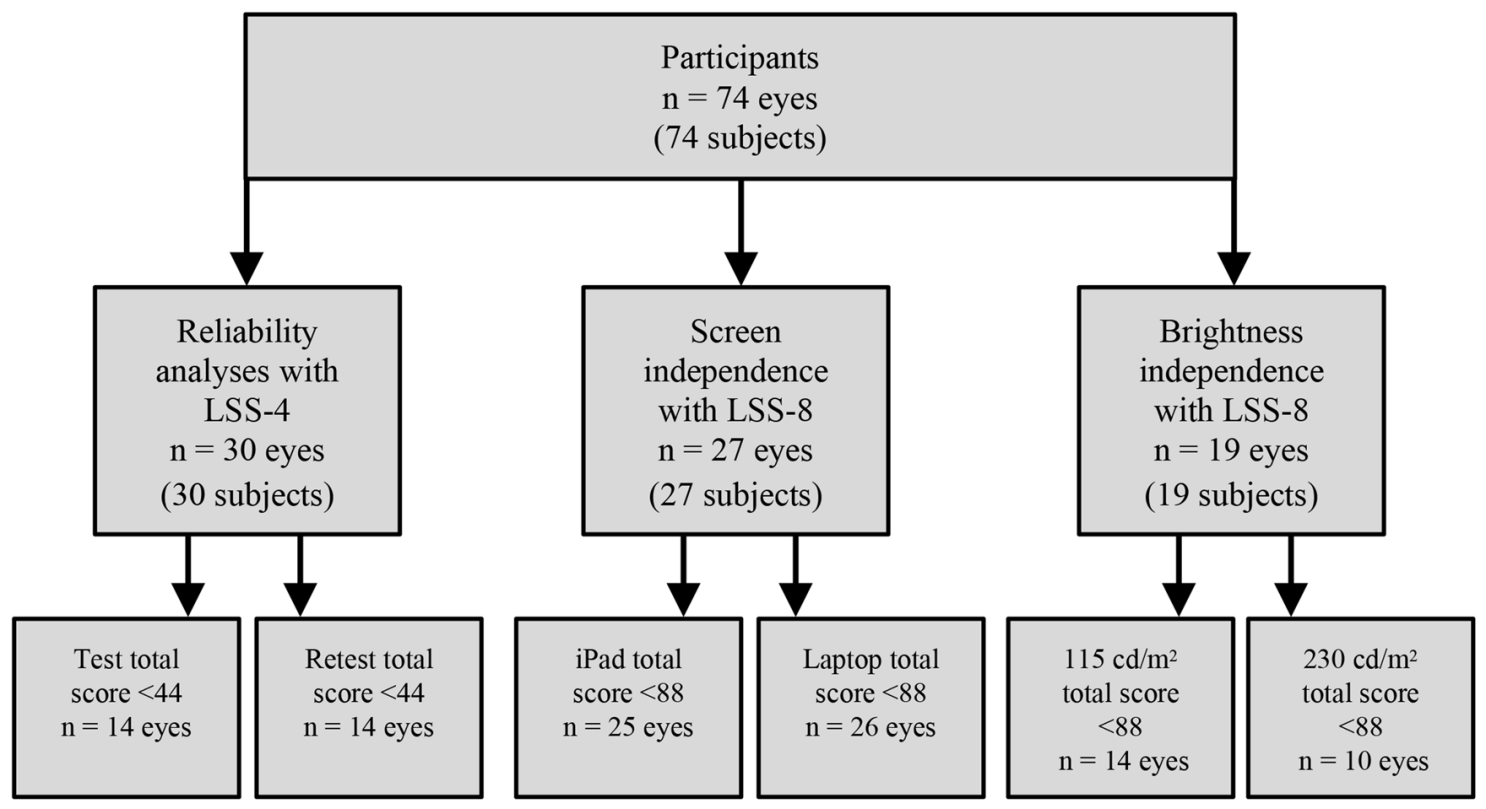
Study participant flow diagram illustrating the three components of the research design and cohort assignment. Two participants contributed to two study phases

Figure 2 illustrates the flow of participants through the three phases of our pilot study. In the test-retest component, 14 participants (47%) did not achieve full marks on the LSS-4. For screen independence, 25 participants (93%) did not score full marks on the iPad test, while 26 participants (96%) did not achieve full marks on the laptop test. Regarding brightness independence, 14 participants (74%) scored below full marks on the low-brightness screen (115 cd/m^2^), while 10 participants (53%) did not achieve full marks on the high-brightness screen (230 cd/m^2^). These data highlight that most included participants did not score full marks on the LSS-4 and LSS-8, supporting that our test has differentiated varying levels of colour vision ability.

### Test-retest reliability

After normalising the plates, we began the reliability phase of our study. Participants were tested with the LSS-4 under the same conditions on different days. Using the ICC(2,1) model, we found that the reliability of all LSS-4 plate sets, as well as the total score, was good to excellent as seen in Table 1. The green plate set showed the lowest ICC(2,1) at 0.87; however, this still represents good reliability. These results confirm that the LSS-4 produces consistent results across different time points, demonstrating its reliability for participants.

**Table 1:** Intraclass correlation coefficient ICC(2,1) for each hue and the total score of hues, for the LSS-4 (top row). ICC(2,k) for iPad versus laptop for the LSS-8 (bottom)

	Mauve	Red	Green	Blue	Pink	Teal	Cobalt	Gold	Total
ICC(2,1)	0.93	0.95	0.87	0.91	NA	NA	NA	NA	0.96
ICC(2,k)	0.93	0.86	0.92	0.82	0.93	0.74	0.81	0.86	0.98

### Screen size and type independence

As seen in Table 1, there is moderate to excellent correlation between the two different screen sizes. Excellent reliability was observed for the mauve, green, pink and total scores, while moderate to good reliability was observed for all other hues. This analysis indicates that participants scored similarly across different screen sizes, reinforcing the consistency of the test.

### Brightness independence

**Fig. 3:**
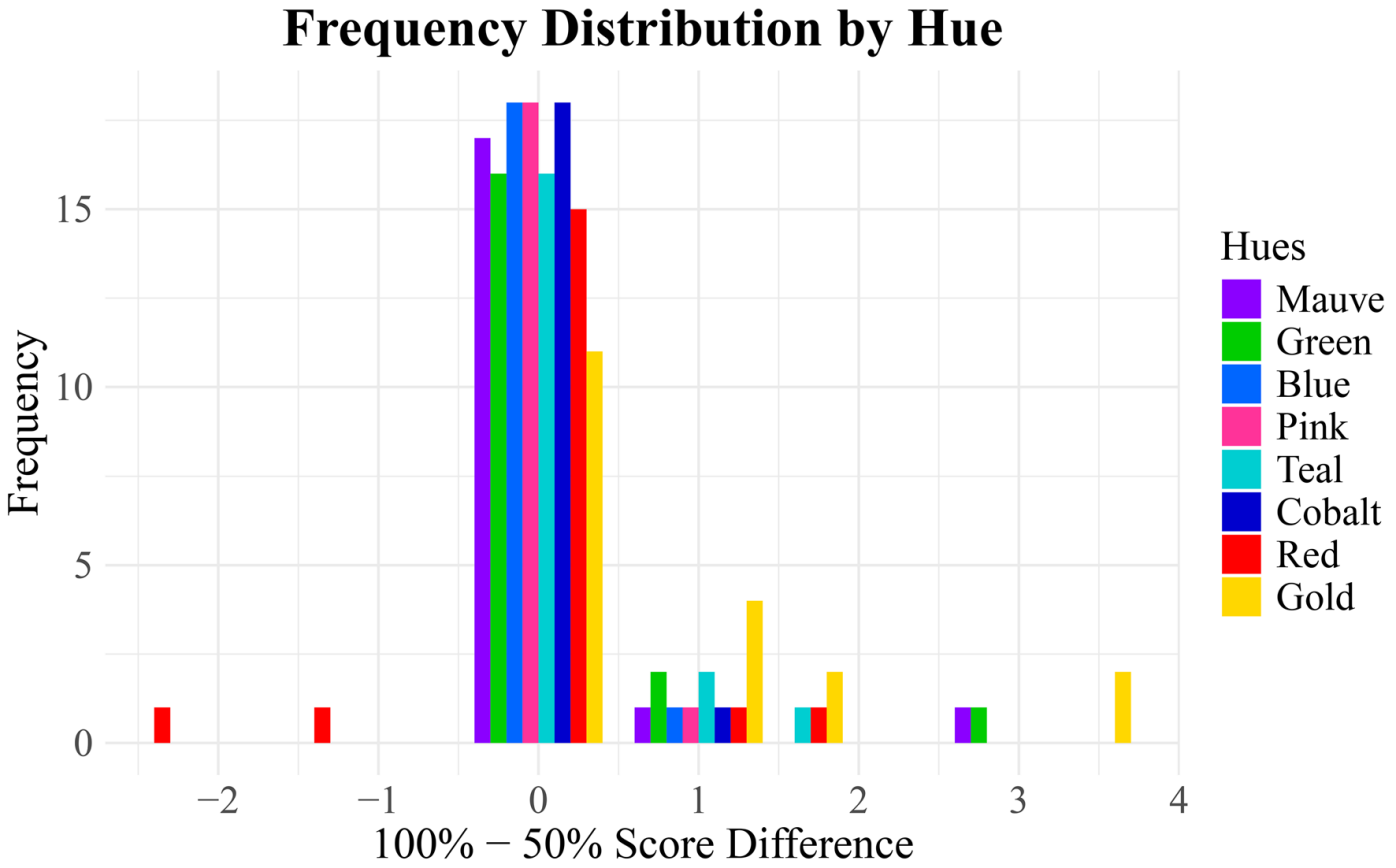
Frequency distribution of score differences between 100% brightness (230 cd/m^2^) and 50% brightness (115 cd/m^2^) across each hue of the LSS-8. The bars show a mostly central distribution for each hue, with few positive and negative outliers. Positive score differences indicate improved performance with increasing brightness

Fig. 3 shows that most participants did not experience a significant score difference between using a screen at 100% brightness (230 cd/m^2^) and at 50% brightness (115 cd/m^2^) on the LSS-8. While we do not intend the test to be used with varying brightness levels, these data suggest that participants scored similarly despite these brightness inconsistencies. We would expect some outliers with positive score differences, indicating that some individuals performed better with increased brightness. Notably, as seen in Fig. 3, there were a few negative score differences, which may be attributed to the randomisation of the testing order, allowing some participants to practice before the low-brightness test.

**Fig. 4:**
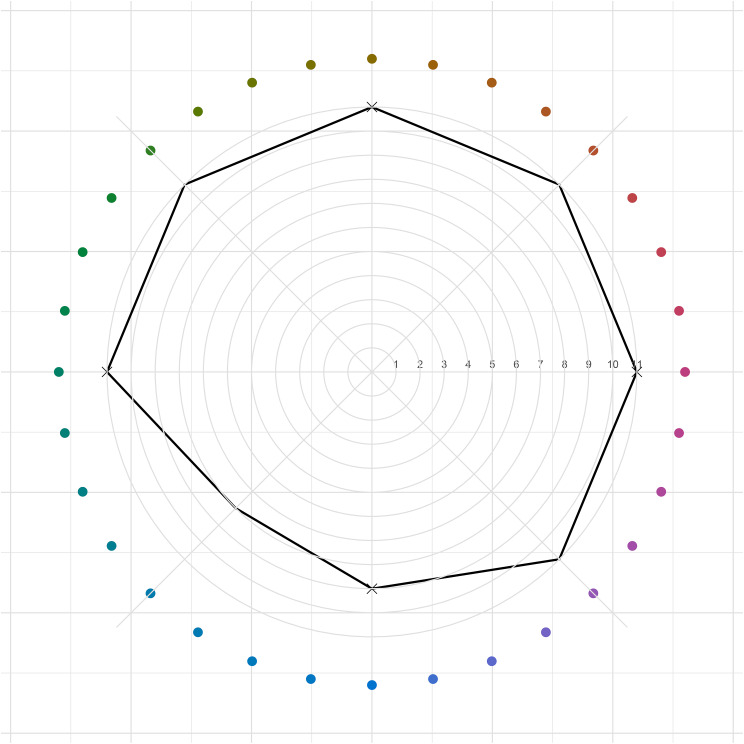
An example of an LSS-8 polar plot where scores out of eleven for each LSS-8 plate hue are graphed to highlight participant deficiencies. In this depiction, the participant scored 11/11 for mauve, red, green, pink, teal and gold. For blue, the polar plot shows an individual scored 8/11, and 9/11 for cobalt, suggesting a defect in these hues

Analogous to the polar plot employed in the FM 100 hue test, LSS-8 scores can be graphically represented, as illustrated in Fig. 4.

## Discussion

The pathophysiology of X-linked congenital colour blindness is well understood, with mutations in the opsin genes of the L- and M-cones causing red-green colour vision deficiencies [44]. These mutations alter the spectral sensitivity of the cones, which in turn affect the relative amplitude of the pathways connecting the cone types to higher visual processing areas. Tests such as the Ishihara pseudoisochromatic plates are widely used to screen for congenital red-green colour vision deficiency [45].

In contrast, the pathophysiology of acquired loss of colour vision is less established in the literature [6]. Disease processes affecting colour vision can occur at various levels of the visual system. For example, pathology may affect the spectral sensitivity or synaptic function of cones. Changes may also occur within bipolar or ganglion cells, impacting their spectral or overall sensitivity. Deviations in ganglion cell discharge patterns, nerve conduction speed or pathology within the brainstem or cortex can affect colour perception [46].

Colour discrimination predominantly occurs within the cortex. Specifically, areas V1, V2, V4 and the inferior temporal cortex have been shown to play a role [47]. The mechanisms for hue and saturation discrimination within the cortex may have different neurological bases [48], with double opponent cells being more important for saturation discrimination and single opponent cells playing a larger role in hue discrimination [49].

The perception of colour forms an important part of the human visual experience. Loss of this function does represent a disability and the ability to measure both hue and saturation discrimination thresholds could be crucial in a comprehensive evaluation of an individual’s visual function – for example, in the evaluation of the effects of medication, illness or injury. Colour deficits may also limit career choices that require accurate colour perception.

Equally, subtle changes in colour vision can be important in the detection of disease induced by medication use, as seen in cases of ethambutol, linezolid and hydroxychloroquine toxicity [31, 50–52]. Tracking disease prognosis with colour saturation threshold testing could assist in evaluating the efficacy of novel treatments for eye disease, such as gene therapy, stem cells or biological agents. This method may also help inform when more aggressive sight-saving treatments are required.

The symptom of relative subjective red desaturation is well known in optic neuritis and is often evaluated through the use of a red bottle top or pin [53]. However, this method provides only a qualitative assessment and cannot track disease progression. A recent study by Nguyen et al. found that Ishihara asymmetry one month post-onset was a strong predictor of visual outcomes and axonal loss in optic neuritis at 6 and 12 months, compared with several other visual measures [54]. We therefore think that the LSS test has great potential utility for monitoring this disease.

The development of colour vision tests has spanned several decades, driven by vocational standards, clinical screening and diagnostic needs [55, 56]. While many tests are available for assessing colour vision, fewer are specifically designed to detect acquired colour vision loss, and many of these are not sensitive enough to detect subtle changes [1, 2, 41]. While the FM 100 hue is well established and validated for hue discrimination, it does not examine the second dimension of saturation discrimination.

In this study, we have developed and validated a novel and freely available test of the saturation threshold of the visual system across multiple hues: The LSS-4 and LSS-8. We anticipate that the LSS-8 will complement the FM 100 hue and may serve as a replacement in specific applications. Our test is similar to the Cambridge Colour test but differs significantly in the spacing of hues in colour space, the representation of the results and, for our test, not requiring any special equipment [57].

Our results suggest our test has sufficient headroom to detect subtle levels of abnormal colour vision, as most participants did not score full marks using the LSS-8. Promisingly, individuals also scored similarly on different days, with varying screen size and display brightness. In clinical application, we do not intend the LSS-8 to be used in this way, however these results indicate the capacity to detect significant clinical deterioration, even using different equipment.

This particular study is relatively limited in size and did not specifically compare the LSS to existing tests of colour vision. Future work will evaluate the relationship of colour defects detected by the LSS to existing tests of colour vision such as the FM 100 hue, Ishihara and Lanthony D15. We also plan to prospectively evaluate the test across a variety of retinal and neuro-ophthalmological conditions. Future studies may also consider the use of age-matched controls as chromatic sensitivity decreases after 40 years, due to a range of factors including an increased optical density of the lens as well as reduced count and efficiency of cones [58–60].

In conclusion, we have presented and shown that the novel LSS-8 is reliable and only minimally dependent on screen type and brightness. Our test is included in the supplementary material accompanying this manuscript and is available for free use under the MIT License. An iOS version is also available through the EyeMate application on the App Store.

## Data Availability

Data are available from the authors upon reasonable request. Please contact the first author, Dr Kristyna Stepnicka, at kristyna.stepnicka@student.unimelb.edu.au.

## Abbreviations

AMD: Age-related macular degeneration
CAD: Colour Assessment and Diagnosis
CIE: Commission Internationale de l'Éclairage
FM 100 hue: Farnsworth-Munsell 100 Hue
HRR: Hardy-Rand-Rittler
ICC: Intraclass correlation coefficient
LCH: Lightness, Chroma, hue
LSS-4: Littlewood-Stepnicka-Sarossy test, 4-plate version
LSS-8: Littlewood-Stepnicka-Sarossy test, 8-plate version
MOG: Myelin oligodendrocyte glycoprotein
NMO: Neuromyelitis optica
